# Biodegraded magnetosomes with reduced size and heating power maintain a persistent activity against intracranial U87-Luc mouse GBM tumors

**DOI:** 10.1186/s12951-019-0555-2

**Published:** 2019-12-23

**Authors:** Edouard Alphandéry, Ahmed Idbaih, Clovis Adam, Jean-Yves Delattre, Charlotte Schmitt, Florence Gazeau, François Guyot, Imène Chebbi

**Affiliations:** 1 Institut de minéralogie et de Physique Des matériaux et de Cosmochimie, UMR 7590 CNRS, Sorbonne Universités, UPMC, University Paris 06, Muséum National D’Histoire Naturelle, 4 Place Jussieu, 75005 Paris, France; 2 Nanobacterie SARL, 36 boulevard Flandrin, 75016 Paris, France; 3 Sorbonne Université, Inserm, CNRS, UMR S 1127, Institut du Cerveau et de la Moelle épinière, ICM, AP-HP, Hôpitaux Universitaires La Pitié Salpêtrière - Charles Foix, Service de Neurologie 2-Mazarin, F-75013 Paris, France; 4 Laboratoire de Neuropathologie, GHU Paris-Sud-Hôpital Bicêtre, 78 rue du Général Leclerc, 94270 Le Kremlin Bicêtre, France; 5 Laboratoire de matière et systèmes Complexes, MSC, Université Paris Diderot, Bâtiment Condorcet, Case 7056, 75205 Paris Cedex 13, France; 6 Institut Universitaire de France, 75013 Paris, France

**Keywords:** Chains of magnetosomes, Magnetotactic bacteria, Glioblastoma, U87-Luc, Antitumor efficacy, Magnetic hyperthermia, Nanomedicine, Oncology, Nanotherapy, Nanooncology, Alternating magnetic field, Thermotherapy, Hyperthermia, Magnetic hyperthermia

## Abstract

**Background:**

An important but rarely addressed question in nano-therapy is to know whether bio-degraded nanoparticles with reduced sizes and weakened heating power are able to maintain sufficient anti-tumor activity to fully eradicate a tumor, hence preventing tumor re-growth. To answer it, we studied magnetosomes, which are nanoparticles synthesized by magnetotactic bacteria with sufficiently large sizes (~ 30 nm on average) to enable a follow-up of nanoparticle sizes/heating power variations under two different altering conditions that do not prevent anti-tumor activity, i.e. in vitro cellular internalization and in vivo intra-tumor stay for more than 30 days.

**Results:**

When magnetosomes are internalized in U87-Luc cells by being incubated with these cells during 24 h in vitro, the dominant magnetosome sizes within the magnetosome size distribution (DMS) and specific absorption rate (SAR) strongly decrease from DMS ~ 40 nm and SAR ~ 1234 W/g_Fe_ before internalization to DMS ~ 11 nm and SAR ~ 57 W/gFe after internalization, a behavior that does not prevent internalized magnetosomes to efficiently destroy U87-Luc cell, i.e. the percentage of U87-Luc living cells incubated with magnetosomes decreases by 25% between before and after alternating magnetic field (AMF) application. When 2 µl of a suspension containing 40 µg of magnetosomes are administered to intracranial U87-Luc tumors of 2 mm^3^ and exposed (or not) to 15 magnetic sessions (MS), each one consisting in 30 min application of an AMF of 27 mT and 198 kHz, DMS and SAR decrease between before and after the 15 MS from ~ 40 nm and ~ 4 W/g_Fe_ down to ~ 29 nm and ~ 0 W/g_Fe_. Although the magnetosome heating power is weakened in vivo, i.e. no measurable tumor temperature increase is observed after the sixth MS, anti-tumor activity remains persistent up to the 15th MS, resulting in full tumor disappearance among 50% of treated mice.

**Conclusion:**

Here, we report sustained magnetosome anti-tumor activity under conditions of significant magnetosome size reduction and complete loss of magnetosome heating power.
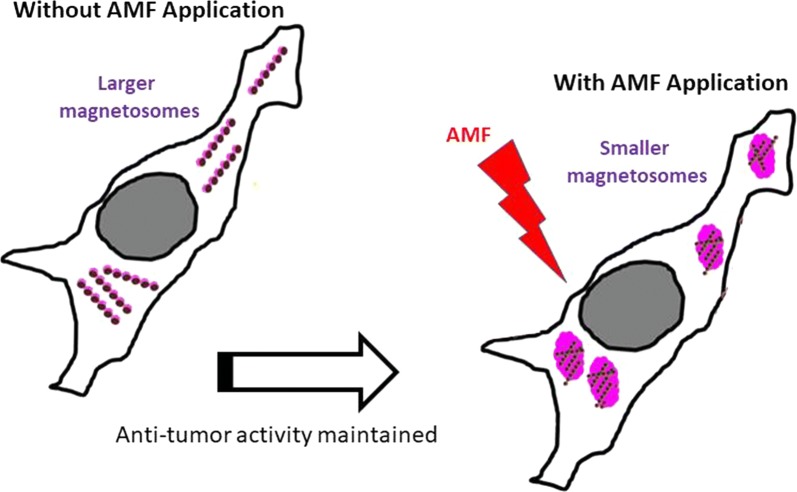

## Background

Cancer thermotherapies currently in use in hospital such as high intensity focused ultrasound necessitate high heating temperatures (typically 80–90 °C) to be efficient, resulting in a number of side effects [1]. To prevent them, the treatment can be carried out at more moderate temperatures or even without any measurable temperature increase by using an external source of energy such as an alternating magnetic field (AMF) applied on nanoparticles contained in a tumor. The latter approach has been tested with various nano-therapeutic systems, leading to encouraging results [2, 3], notably in the treatment of glioblastoma by magnetic hyperthermia [4, 5]. In order to be optimal, such treatments necessitate that nanoparticles remain for a sufficiently long time in the tumor to induce strong and persistent anti-tumor activity until full tumor disappearance. At the same time, nanoparticles also need to be eliminated. Their long-term accumulation in a specific part of the organism should be avoided. Such a fine adjustment of nanoparticle bio-distribution properties can be obtained by using nanoparticles that are progressively captured and degraded by the organism [6, 7]. However, such behavior is often associated with a reduction in size, crystallinity, heating power, and anti-tumor efficacy of nanoparticles [8]. To the author knowledge, it has not yet been shown that nanoparticles could remain efficient against a tumor after significant alteration.

Here, we study a certain type of nanoparticles, called magnetosomes, which are synthesized by magnetotactic bacteria (AMB-1) and are composed of a mineral iron oxide core surrounded by a layer consisting of biological material, mainly consisting of lipids, proteins, and lipopolysaccharides [9–16]. Anti-tumor activity has been demonstrated upon injection of magnetosomes and application of alternating magnetic fields [14–17]. Magnetosomes possess a well-balanced size distribution with 58% of them larger than 30 nm and 42% of them smaller than 30 nm (Table 1) [10]. This size distribution enables the observation of changes in magnetosome sizes under various conditions of alteration in vitro and in vivo. Here, we study whether altered magnetosomes can yield anti-tumor activity both in vitro by examining if magnetosomes internalized in U87-Luc tumor cells can destroy these cells and in vivo by monitoring the decrease in bioluminescence intensity (BLI) emitted by U87-Luc tumors implanted in mouse brains, which are injected with magnetosomes and exposed to 15 sessions of alternating magnetic field application. Furthermore, we also examine the link between magnetosome size alteration, anti-tumor activity, and weakening of heating power.

**Table 1:** Percentages of magnetosomes smaller than 30 nm, percentages of magnetosomes larger than 30 nm, and dominant magnetosome sizes within the whole magnetosome size distribution for untreated magnetosomes, magnetosomes internalized in U87-Luc cells, magnetosomes administered to intracranial U87-Luc tumors without magnetic treatment, or magnetosomes injected to intracranial U87-Luc tumors followed by 15 magnetic sessions during which an AMF of 198 kHz and 27 mT is applied during 30 min

	Percentage of magnetosomes smaller than 30 nm	Percentage of magnetosomes larger than 30 nm	Dominant magnetosomes size DMS (nm)
Untreated magnetosomes	42	58	40
Internalized magnetosomes (in vitro)	92.5	7.5	11
Magnetosomes administered in U87-Luc tumors and kept in the tumors for 30 days without AMF application	23.5	76.5	43
Magnetosomes administered in intracranial U87-Luc tumors followed by 15 MS	64	36	29

## Results and discussion

### Magnetosome properties (size distribution, heating power, and endotoxin release) prior to their administration

**Fig. 1:**
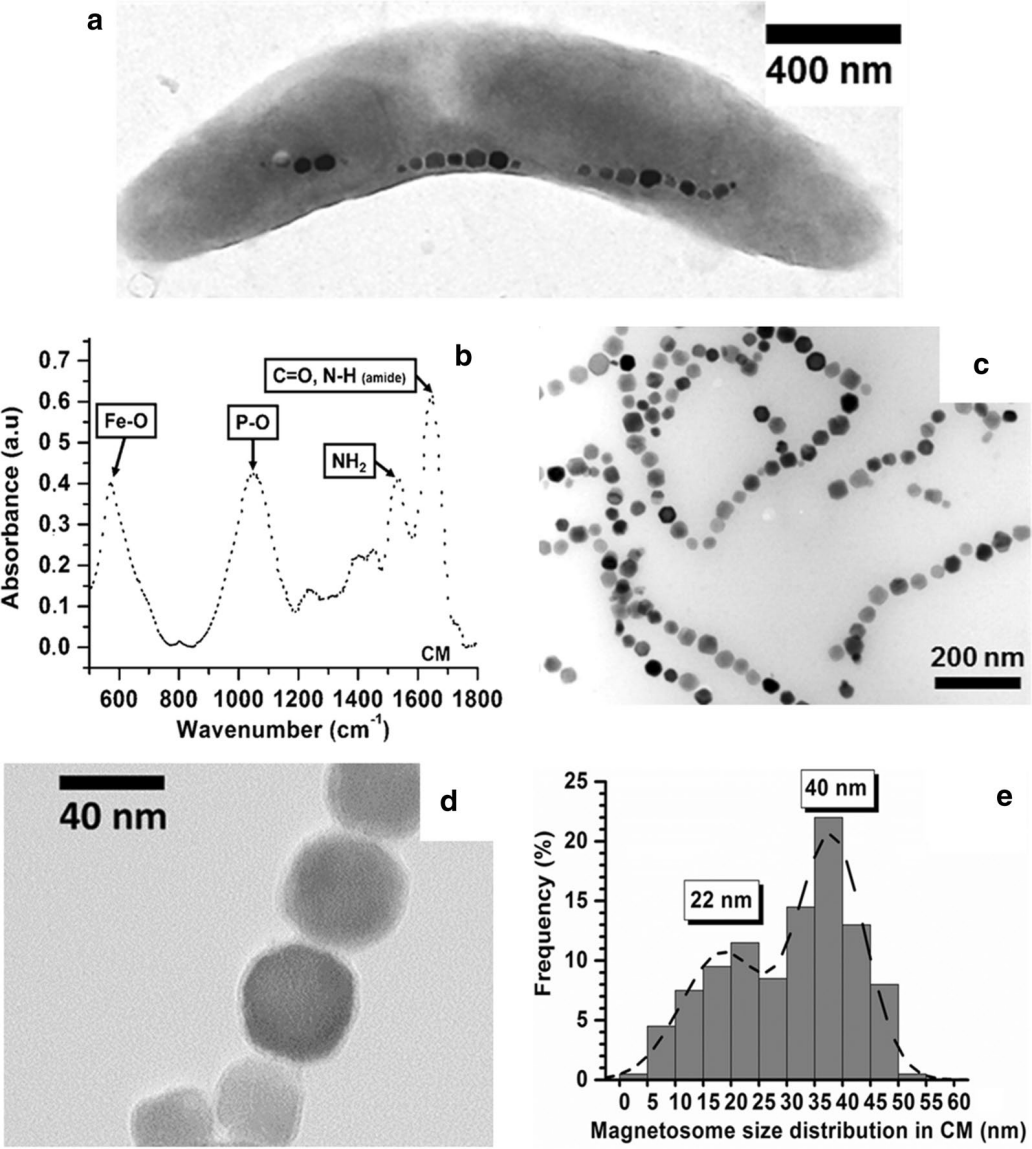
**a** TEM image of a whole magnetotactic bacterium, showing several chains of magnetosomes inside the bacterium. **b** FT-IR spectrum of chains of magnetosomes. **c**, **d** TEM images of chains of magnetosomes extracted from magnetotactic bacteria (CM). **e** size histogram of magnetosomes shown in **c** estimated over 350 magnetosomes

Here, we study the properties of nanoparticles, called magnetosomes, which are synthesized by magnetotactic bacteria, of which a typical transmission electron microscopy (TEM) image is presented in Fig. 1a.

The preparation of the injectable magnetosome suspension involved the following steps: (i), growth of AMB-1 *Magnetospirillum magnetotacticum* magnetotactic bacteria (ATCC 700264) during 7 days, (ii), harvesting of a concentrated pellet of these bacteria, (iii), lysis of these bacteria under sonication at 0 °C during 2 h at 30 W, (iv), isolation of magnetosome chains (CM) from cellular organic debris using a magnet, (v), re-suspension of CM in a sterile injectable solution containing 5% of glucose, and (vi), partial sterilization of the CM suspension by exposing CM suspension to UV irradiation for 12 h.

The stability of CM in suspension, which is required to carry out in vitro and in vivo studies, is revealed, firstly by the behavior of the zeta potential variation of this suspension as a function of pH that displays a well-defined and repeatable behavior, i.e*.* a decrease from 20 mV at pH 2 to − 35 mV at pH 12 (Additional file 1: Figure S1a), and secondly by the optical absorption of this suspension, measured at 480 nm, which decreases moderately, i.e. by less than 30% within 20 min following homogenization of this suspension (data not shown). Magnetosome composition was reported to consist of an iron oxide mineral core surrounded by biological material, containing endotoxins made of lipopolysaccharides (LPS), which binds magnetosomes together [18, 19]. On the one hand, such composition was deduced from infra-red absorption measurements carried out on a lyophilized suspension of CM, denatured and dispersed in KBr, whose infra-red absorption spectra displayed (Fig. 1b): (i), Amide I and Amide II bands due to protein absorption at 1650 cm^−1^ and 1530 cm^−1^ [20–22], (ii), absorption bands due to lipopolysaccharide (LPS) or phospholipids contained in the magnetosome membrane at 1050 cm^−1^ and 1250 cm^−1^ [20, 21], (iii), a peak at 580 cm^−1^ attributed to maghemite [22], as reported elsewhere [22]. CHNS elemental analysis of CM revealed a percentage of 13.9% of carbonaceous material surrounding the magnetosome mineral core (Additional file 1: Figure S1b). Furthermore, part of the organic material surrounding the iron oxide mineral core of the magnetosomes is composed of endotoxins, as highlighted by limulus amebocyte lysate (LAL) measurements carried out on a CM suspension that shows the presence of 2000 EU of endotoxins per mg in iron per ml of CM suspension. Magnetosome size distributions are measured by analysis of TEM images of a dried suspension of CM deposited on top of a carbon-coated TEM grid, such as those presented in Fig. 1c, d. The distribution displays two peaks centered at 22 ± 4.6 nm and 40 ± 2.7 nm with a mean size at 37.5 ± 5.2 nm (Fig. 1e), resulting in nanoparticles of sufficiently large sizes to yield a ferrimagnetic behavior at room temperature, i.e. with H_C_ ~ 20 mT and M_r_/M_S_ ~ 0.3 (Additional file 1: Figure S1c), and therefore also strong heating power (Additional file 1: Figure S1d) [9]. The latter was measured for suspensions of magnetosomes in water under two conditions: 2 mg of magnetosomes contained in 100 µl of water (condition 1) and 40 µg of magnetosomes suspended in 2 µl of water (condition 2), the same quantities of magnetosomes as those used for in vitro and in vivo experiments, which were exposed to the one MS, i.e. the application of an AMF of 198 kHz and average strength 27 mT during 600 s. The SAR was estimated using the formula SAR = (ΔT/δt)·(C_v_/X_Fe_), where C_v_ = 4.2 J/gK is the specific heat capacity of water, X_Fe_ = 20 mg/ml (conditions 1 and 2), ΔT/δt = 5.9 ± 1.5 °C/s (condition 1) and ΔT/δt = 9.5 × 10^−3^ ± 1.8 × 10^−3^ °C/s (condition 2). Interestingly, the first condition of treatment yielded much larger SAR and temperature increase over the whole MS (ΔT), i.e. SAR = 1234 ± 307 W/g_Fe_ and ΔT = 95 ± 8 °C, than the second one, i.e. SAR = 57 ± 6 W/g_Fe_ and ΔT = 33 ± 3 °C (Table 2). These results suggest that for an equivalent magnetosome concentration (here 20 µg/µl), magnetosome heating power decreases with decreasing volume comprising magnetosomes. Although such behavior is rarely reported in the literature, possibly due to the use of adiabatic heating conditions that are far from the real in vivo heating environment, it could be due to enhanced heat diffusion between the interior and exterior of the magnetosome suspension, which is willingly contained in a non-adiabatic Eppendorf, due to larger surface to volume ratio when magnetosomes are contained in a smaller volume. This result suggests a mechanism by which magnetosome heating power is improved when magnetosomes become distributed within a larger volume, a situation that may occur in vivo when magnetosomes distribution becomes scattered within the whole tumor over time. Since magnetosome anti-tumor activity could come not only from a purely thermal effect but also from an immune reaction triggered by endotoxin release, the effect of the application of the same MS as above on magnetosome endotoxin release was studied. Interestingly, magnetosomes continue to release endotoxins after AMF application. Indeed, moderate endotoxin release is measured in the supernate of suspensions of magnetosomes following one MS. Endotoxin release is estimated as Q_AMS_/Q_BMS_ ~ 0.5%, where Q_AMS_ and Q_BMS_ are the quantities of endotoxins contained in the supernate of a CM suspension after and before one MS for Q_AMS_ and Q_BMS_, respectively.

**Table 2:** Initial slope of the temperature variation with time, ΔT/δt, SAR deduced using the formula SAR = (ΔT/δt)⋅(Cv/X_Fe_), where C_v_ = 4.2 J/gK is the specific heat capacity of water and X_Fe_ is the magnetosome concentration, and temperature increase measured over the whole magnetic session, ΔT. ΔT/δt, SAR and ΔT were measured for magnetosomes suspended in water (2 mg of magnetosomes in 100 µl of water or 40 µg of magnetosomes in 2 µl of water), magnetosomes after cellular internalization in vitro, and magnetosomes administered to U87-Luc intracranial tumors and exposed to one MS (MS1), two MS (MS2), three MS (MS3), four MS (MS4), five MS (MS5), six to fifteen MS (MS6 to MS15)

	Magnetosome in water suspension (2 mg/100 μl)	Magnetosome in water suspension (40 μg/2 μl)	Magnetosome incubated with U87-Luc cells during 24 h	MS1 (in vivo)	MS2 in vivo	MS3 in vivo	MS4 in vivo	MS5 in vivo	MS6 to MS15 in vivo
∆T/δt (°C/s	5.9 ± 1.5	0.27 ± 2.8×10^−2^	9.5 × 10^−3^ ± 1.8 × 10^−3^	2.2 × 10^−2^ ± 7.1 × 10^−3^	1.1 × 10^−2^ ± 4.4 × 10^−3^	1.1 × 10^−5^ ± 5.5 × 10^−7^	1.1 × 10^−5^ ± 5.5 × 10^−7^	1.1 × 10^−5^ ± 5.5 × 10^−7^	0
SAR (W/gFe)	1234 ± 307	57 ± 6	57 ± 11	4.7 ± 1.5	2.5 ± 1	2 × 10^−3^ ± 10^−4^	2 × 10^−3^ ± 10^−4^	2 × 10^−3^ ± 10^−4^	0
∆T (°C)	95 ± 8	33 ± 3	7.4 ± 0.7	4 ± 1	1.7 ± 1	0.4 ± 0.4	0.4 ± 0.4	0.4 ± 0.4	0

### Magnetosome internalization inside U87-Luc cells results in a decrease of magnetosome sizes, heating power, while maintaining a faculty to destroy these cells

**Fig. 2:**
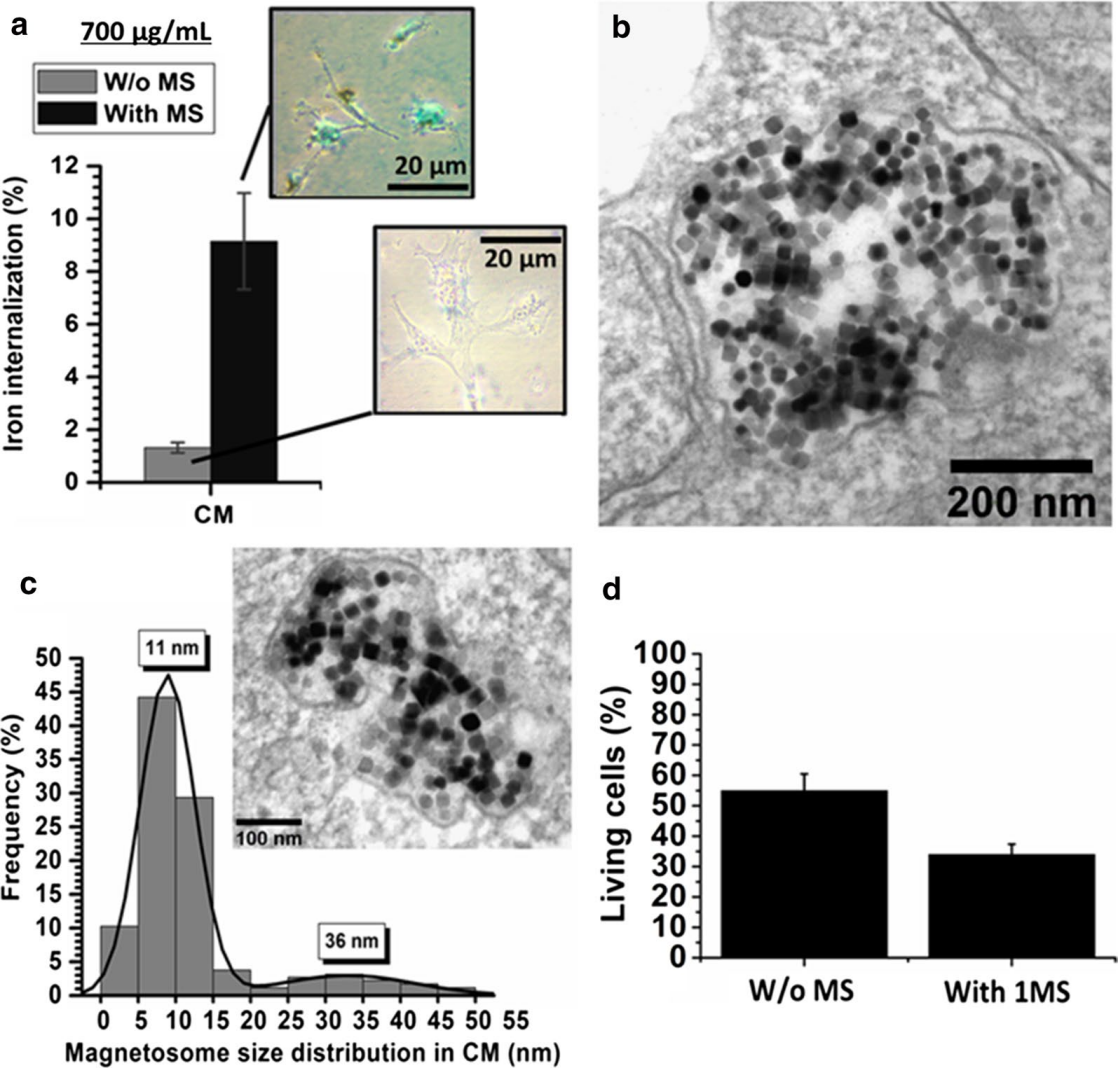
**a** Percentage of iron internalized in U87-Luc cells measured by ICP-AES, when these cells were brought into contact with 700 µg/ml of CM during 24 h, and either not subjected to one MS (w/o MS) or subjected to one MS (with MS). In the inset of **a**, optical microscopy images of cells brought into contact with CM and stained with Prussian blue. **b**, **c** TEM images and associated size histogram estimated on 150 magnetosomes of magnetosomes internalized in U87-Luc cells, within an intracellular vesicle, following the treatment described in **a**. **d** Percentage of living cells measured following a treatment in which U87-Luc cells were brought into contact with 700 µg/ml of CM during 24 h and either no exposed to one MS (W/o MS) or exposed to one MS (With 1 MS). The MS consisted in the application of an AMF of 198 kHz and 27 mT during 30 min

Next, we study the effect of magnetosome cellular internalization on magnetosome sizes, heating power, and anti-tumor activity. For that, 2 ml of suspensions of magnetosome, containing 1.4 mg of iron, were incubated with U87-Luc cells during 24 h followed (or not) by one MS during which an AMF of 27 mT and 198 kHz was applied during 30 min. To verify that such treatment resulted in magnetosome cellular internalization, we carried out optical microscopic observations of assemblies of cells and magnetosomes stained with Prussian blue. They reveal a cyan coloration at cell location, which is more persistent after than before one MS (inset of Fig. 2a), suggesting that magnetosome internalization is enhanced following one MS. Such behavior is further supported by estimating the percentage of iron internalized in cells, using ICP-AES iron dosage carried out on these assemblies dissolved by Nitric acid. It increases from 1.5% before one MS to 9.2% after one MS. To examine more precisely magnetosome location following internalization, the cells brought into contact with magnetosomes were cut with a diamond knife in an ultra-microtome in 80 nm thin slices. The latter were deposited on top of a carbon-coated TEM grid and examined by TEM. Under these conditions of treatment, the TEM images of Fig. 2b, c show that magnetosomes are localized inside a cellular vesicle of 0.2–0.5 µm, which is most probably a lysosome or an endosome. The effect of cellular degradation on size and organization of magnetosomes can also be observed in these electron microscopy images. Indeed, compared with magnetosomes of Fig. 1c that were not treated, i.e. kept in water suspension, those of Fig. 2b, c appear to have lost their organization in chains, to have acquired for some of them a different shape, as observed in Fig. 2b, c, and to have become smaller. Indeed, their size distribution shows that 92.5% of them are below 30 nm while 7.5% of them are above 30 nm (Table 1). The size distribution displays two peaks centered at 11 ± 1.8 nm and 36 ± 8.6 nm with a mean size at 12 ± 7.9 nm (Fig. 2c). These observations indicate a magnetosome degradation following cellular internalization. Furthermore, this intracellular degradation is accompanied by a loss of heating power, as revealed by the measurements of SAR and ΔT, which decrease from SAR = 1234 ± 307 W/g_Fe_ and ΔT = 95 ± 8 °C before internalization to SAR = 57 ± 11 W/g_Fe_ and ΔT = 7.4 ± 0.7 °C following internalization, where the latter SAR value was estimated using the formula SAR = (ΔT/δt)·(Cv/X_Fe_), where C_v_ = 4.2 J/gK, X_Fe_ = 0.7 mg/ml, and ΔT/δt = 9.5 ± 1.8 °C/s (Table 2). Most interestingly, despite this alteration, the application of the magnetic field on magnetosome is accompanied by a decrease in the percentage of living cells from ~ 55% without MS to ~ 30% with one MS (Fig. 2d). Although the magnetosome heating power is weakened in vitro, it remains significant and could cause the death of tumor cells. Another reason for the observed cytotoxicity could be endotoxin release that might result from the process of magnetosome degradation inside the cells. Such mechanism is not directly proven here, since endotoxin release would be difficult to monitor inside the cells. Instead, it is suggested based on previous reports describing endotoxins as being able to cause cellular death [23, 24].

### Magnetosomes administered to intracranial U87-Luc glioblastoma tumors and exposed to 15 sessions of alternating magnetic field application reduce in sizes and heating power, while keeping efficient anti-tumor activity resulting in full tumor disappearance among 50% of treated mice

**Fig. 3:**
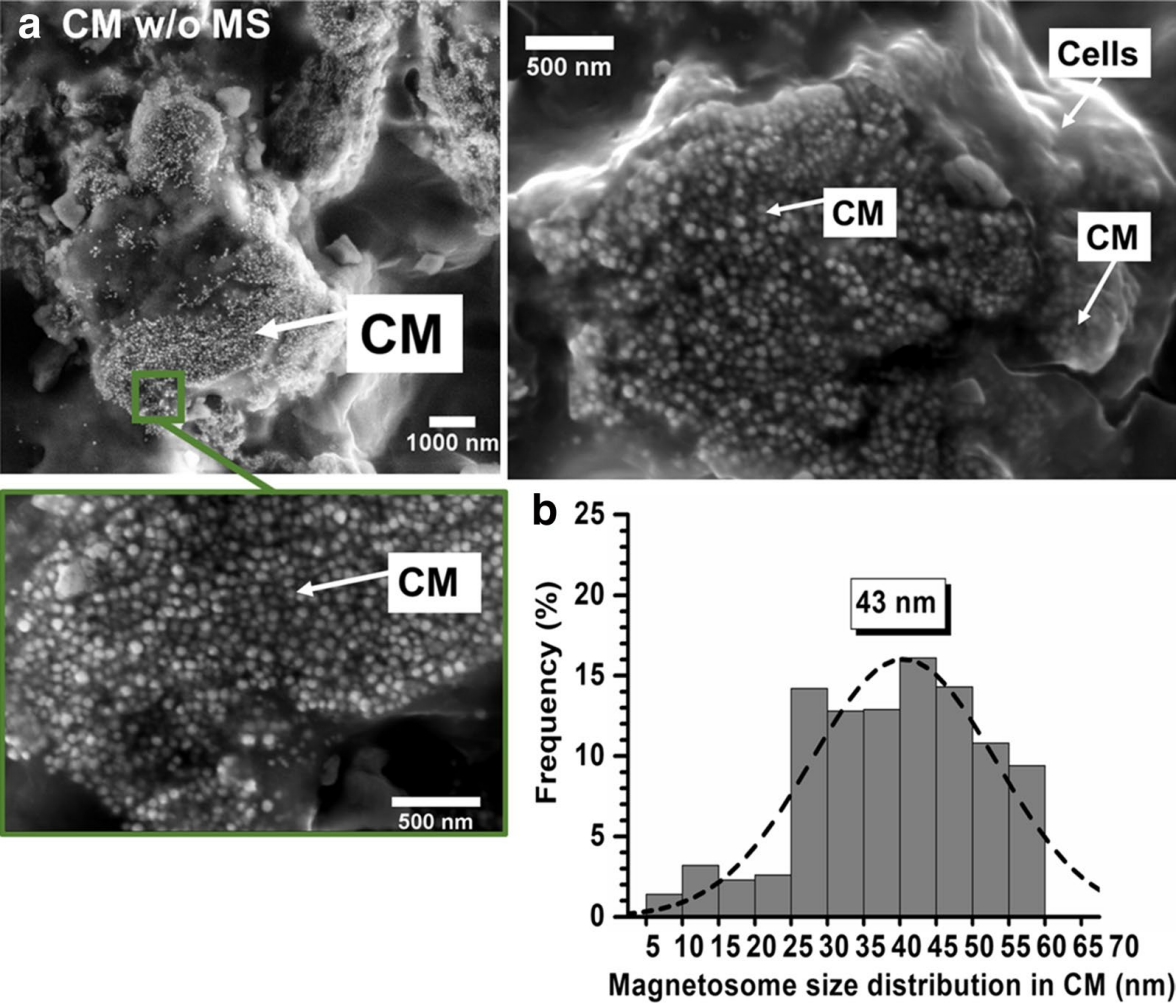
**a** Scanning electron microscopy (SEM) images of a brain section collected at D30 from a mouse treated by CM administration, showing co-localization of cells and CM. **b** Magnetosome size distribution deduced from **a**, estimated from the size of 600 magnetosomes

The impact of intra-tumor magnetosome administration on nanoparticle size is examined. For that, slides of brains of mice bearing U87-Luc tumors treated by CM administration without MS are collected 30 days following MC administration and analyzed by scanning electron microscopy (SEM). The SEM image of a slide treated in these conditions is presented in Fig. 3a. It shows that magnetosomes are localized in the same region as cells and are characterized by an average size of ~ 43 ± 8 nm (Fig. 3b). Compared with the size distribution of magnetosomes before administration (Fig. 1e), that of Fig. 3b has become mono-modal, the proportion of the largest magnetosomes has increased, i.e. 76.5% of magnetosomes are larger than 30 nm, whereas the percentage of the smallest ones has decreased, i.e. 23.5% of magnetosomes are smaller than 30 nm (Table 1), suggesting that magnetosome intra-tumor administration resulted in a preferential dissolution of the smallest magnetosomes.

**Fig. 4:**
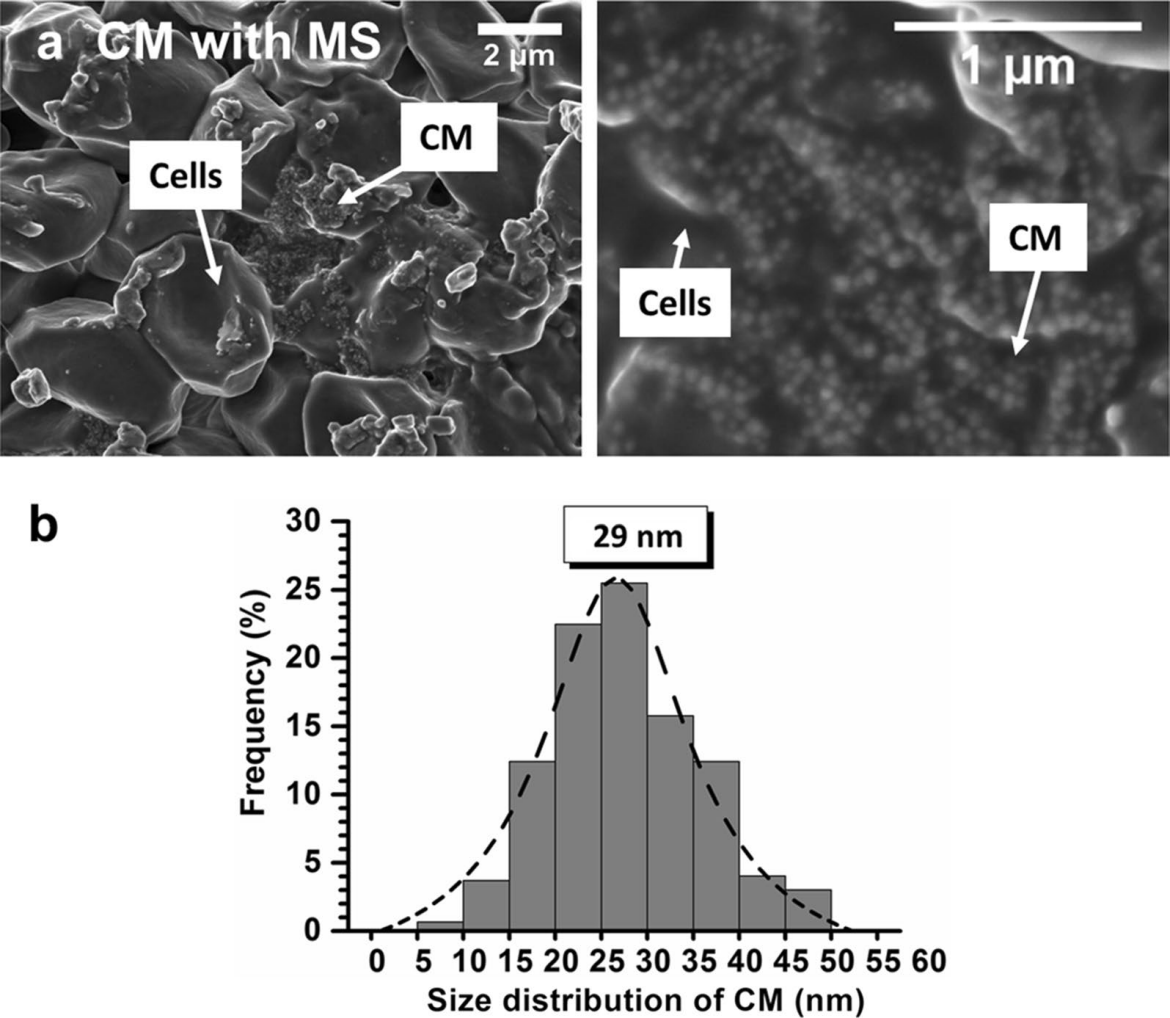
**a** Scanning electron microscopy (SEM) images of a brain section collected at D30 from a mouse treated by CM administration followed by 15 MS, where each MS consisted in the application of an AMF of 198 kHz and 27 mT applied during 30 min, showing co-localization of cells and CM. **b** Magnetosome size distribution deduced from **a**, estimated from the size of 600 magnetosomes

To reflect the conditions of magnetic hyperthermia treatment, magnetosomes were then treated by the previously mentioned step of intra-tumor CM administration, which was followed by 15 magnetic sessions of alternating magnetic field application. Slides of mouse brains treated under these conditions were examined by SEM. They reveal the presence of CM co-localized with cells (Fig. 4a) as in Fig. 3a, but with an average magnetosome size that has decreased down to ~ 29 ± 8 nm (Fig. 4b). Compared with the treatment without MS, that with MS led to a percentage of the smallest magnetosomes that is larger, i.e. ~ 64% of magnetosomes are smaller than 30 nm, and to a percentage of the largest magnetosomes that is smaller, i.e. ~ 36% of magnetosomes are larger than 30 nm (Table 1), suggesting more stringent conditions of degradation with than without MS, leading to the partial dissolution of the largest magnetosomes. In addition, while in vitro size-measurements carried out on internalized magnetosomes showed that more than 80% of magnetosomes are between 1 and 15 nm (Fig. 2c), the histogram of Fig. 4b indicates that this range of magnetosome sizes occurs for less than 5% of magnetosomes following in vivo treatment, which might suggest that magnetosome alteration is different in vitro and in vivo.

Although magnetosome alteration is expected to undermine magnetosome heating power, it does not necessarily deprive magnetosomes of the faculty to destroy the tumor, as previously described during the analysis of the in vitro results. For this reason, we have studied magnetosome in vivo anti-tumor activity by using 60 nude mice, which were divided into 6 different groups of 10 mice each. We first administered 10^5^ U87-Luc cells inside the brains of these mice at the injection site of (0.2.0) bregma coordinates using a stereotactic helmet. We waited for 1 week that the tumor reached 2 mm^3^ and started the treatment at D8 (8 days following tumor cell administration at D0). The groups were treated as follows (Additional file 1: Table S3):

- Group 1 received at D8 2 µl of a solution of 5% of glucose at (0.2.0);
- Group 2 received at D8 2 µl of 5% of glucose at (0.2.0) followed by 3 MS at D8, D9, and D10;
- Group 3 received at D8 2 µl of 5% glucose at (0.2.0) followed by 12 MS at D8, D9, D10, D15, D16, D17, D22, D23, D24, D29, D30, D31;
- Group 4 received at D8 2 µl of 40 µg of CM at (0.2.0);
- Group 5 received at D8 2 µl of 40 µg of CM at (0.2.0) followed by 3 MS at D8, D9, D10;
- Group 6 received at D8 2 µl of 40 µg of CM at (0.2.0) followed by 15 MS at D8, D9, D10, D15, D16, D17, D22, D23, D24, D29, D30, D31, D36, D37, D38;

Each MS consisted in the application of an AMF of average strength 27 mT and frequency 198 kHz during 30 min. During the course of each MS, the temperature of the mouse brain was monitored with an infra-red camera. The size of the tumor, which was shown to be proportional to the tumor bioluminescence intensity BLI [25], was followed by measuring the BLI of the mouse brains in-between the various MS.

**Fig. 5:**
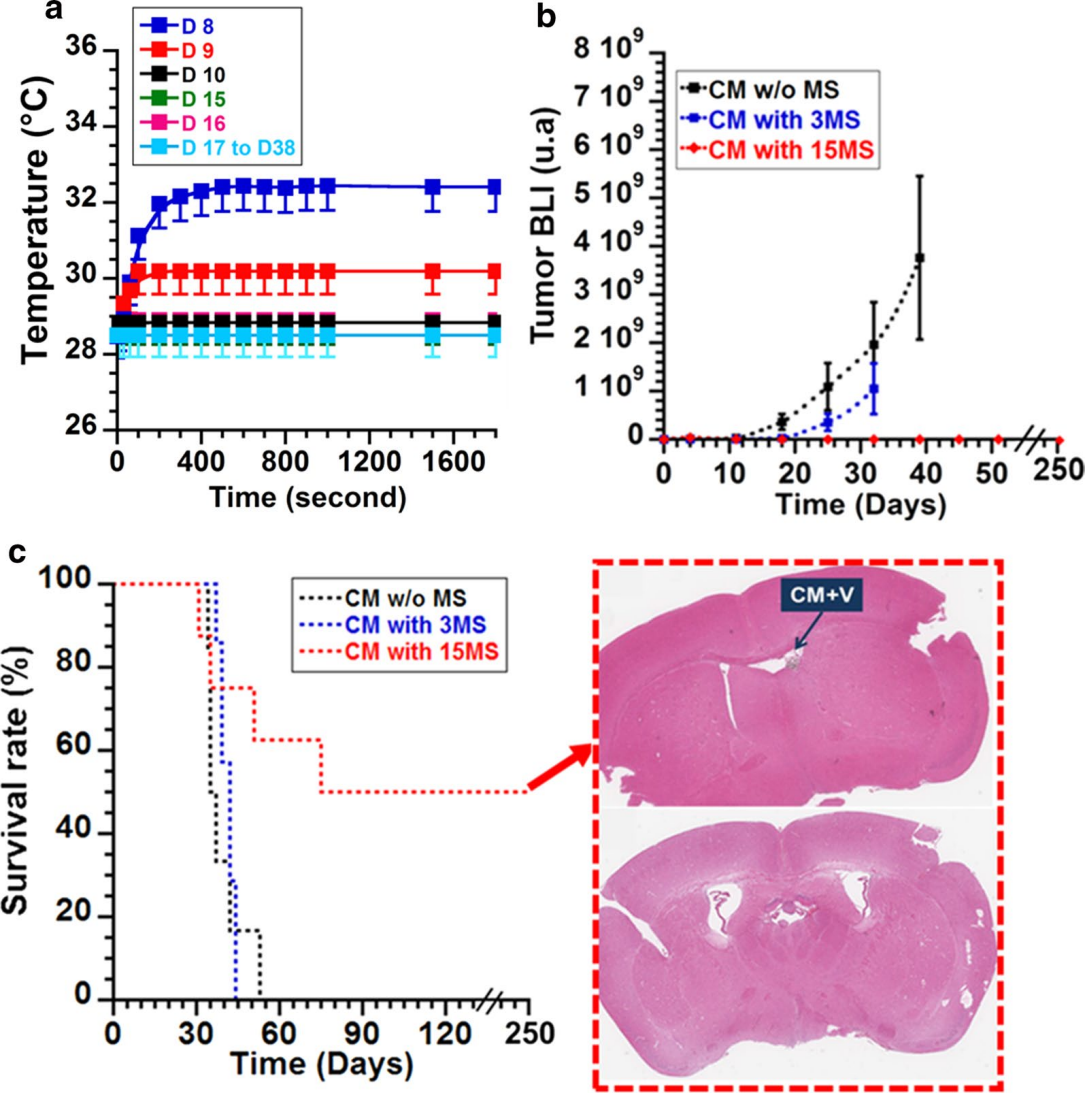
For a mouse having received CM followed by 15 MS, temperature variation measured with an infra-red camera during the course of each MS (**a**). For mice having received CM without MS or CM followed by 3 or 15 MS, variation of tumor BLI (**b**), survival rate (**c**), during the days following tumor cell implantation. The inset of **c** shows two representative histological images of brain slides of a mouse euthanized 250 days following tumor cell implantation. Both slides show an absence of tumor. One image shows the presence of CM while the other one lacks CM. CM + V designates chains of magnetosomes in ventricles

We have measured tumor temperature increases during the various MS of group 6 treated by CM administration followed by 15 MS. During the first 5 MS, the tumor temperature increased by 4 ± 1 °C (MS1), 1.7 ± 1 °C (MS2), 0.4 ± 0.4 °C (MS3 to MS5), resulting in SAR of 4.7 ± 1.5 W/gFe (MS1), 2.5 ± 1 W/gFe (MS2), 2 × 10^−3^ ± 10^−4^ W/gFe (MS3 to MS5), where the SAR is measured using the formula SAR = (ΔT/δt)·(C_v_/X_Fe_), where Cv = 4.2 J/gK is the specific heat capacity of water, X_Fe_ = 0.02 g/ml is the nanoparticle concentration in iron, (ΔT/δt) is the initial slope of the temperature variation with time deduced from Fig. 5a as ΔT/δt = 2.2 × 10^−2^ ± 7.1 × 10^−3^ °C/s (MS1), ΔT/δt = 1.1 × 10^−2^ ± 4.4 × 10^−3^ °C/s (MS2), ΔT/δt = 1.1 × 10^−5^ ± 5.5 × 10^−7^ °C/s (MS3 to MS5). Compared with the SAR measured in solution for 40 µg of CM suspended in 2 µl of water (SAR = 57 ± 6 W/g_Fe_), the SAR measured in vivo is lower during the different sessions, which could be explained on the one hand by the diffusion of magnetosomes outside the injection volume and on the other hand by the loss of the Brownian contribution to the heating mechanism, due to a weaker magnetosome movement in tissue than in water. Furthermore, the decrease of the magnetosome heating power between MS1 and MS5, which is followed by a non-measurable heating of the tumor between MS6 and MS15, could come from the decrease of magnetosome sizes following the various MS, as highlighted by the SEM analyses of magnetosomes administered to U87-Luc tumors and exposed to 15 MS (Fig. 4b).

**Fig. 6:**
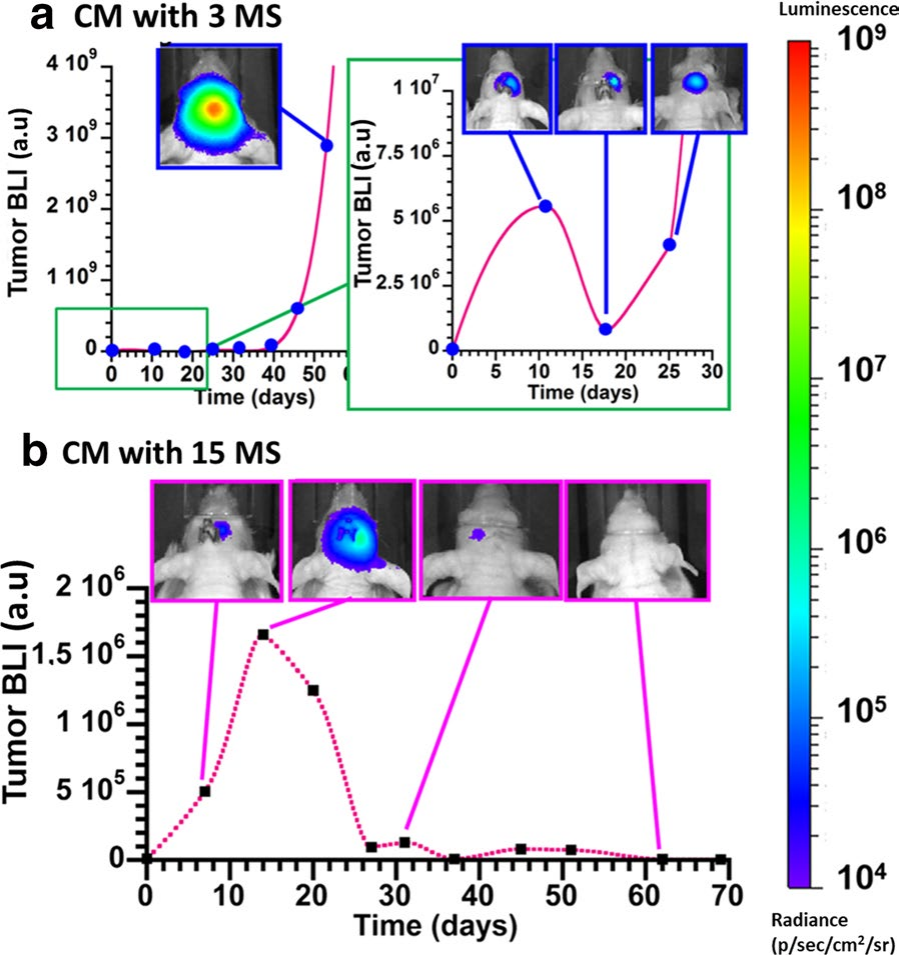
For two mice having received CM followed by 3 MS (**a**), or 15 MS (**b**), variation of tumor BLI as a function of time (days) following tumor cell implantation. The insets of **a** and **b** show optical images of the brains with an indication of brain BLI

We have then examined the effect of the treatment on anti-tumor activity when magnetosomes were altered following intra-tumor administration and the various MS. In control groups, which received glucose with/without MS (groups 1, 2, 3), or CM without MS (groups 4), tumor BLI increased exponentially from 0 at D0 to 1–7 10^9^ at D35 (Additional file 1: Figure S2a), the temperature of the mouse brain remained constant at 29 °C during the course of each MS (Additional file 1: Figure S2b), and anti-tumor activity did not take place. Mice belonging to these groups were euthanized at D37 to D44 (Additional file 1: Figure S2c), when the tumor bioluminescence intensity exceeded 9 × 10^9^. By contrast, when mice belonging to group 5 were injected with CM followed by 3 MS, it resulted in partial anti-tumor activity, as highlighted by the behavior of the BLI averaged over all mice that increased more slowly in group 5 than in group 4 (Fig. 5b) and by the BLI of a typical mouse belonging to group 5 that decreases between D11 and D18 (Fig. 6a). In this mouse, such activity is insufficient to prevent tumor re-growth after D18, and the tumor BLI exponentially increases between D18 and D25, as observed in the inset of Fig. 6a. Mice of group 5 needed to be euthanized at D42 (Fig. 5c), without an improvement of the median survival day compared with control group 4 (Fig. 5c). In order to further enhance therapeutic activity, mice belonging to group 6 were injected with CM and exposed to an additional 12 MS as compared with group 5. Under these conditions, the improvement of therapeutic activity is revealed firstly by the BLI averaged over all mice that does not increase between D0 and D250 (Fig. 5b), secondly by the BLI of a mouse of group 6, which continuously decreases between D12 (after 3 MS) and D38 (after 15 MS) and remains almost undetectable after D38 (Fig. 6b), thirdly by the full tumor disappearance in 50% of mice belonging to this group, which are still alive at D250 (Fig. 5c), and fourthly by a mouse median survival day (MSD) above D250, which is much larger than the MSD of D36 to D42 estimated for the other groups (Additional file 1: Table S3). Furthermore, mice, which were still alive at D250, were euthanized for histological analysis. Optical micrographs of two representative brain sections of these mice are presented in Fig. 5c, showing either some remains of magnetosomes or no sign of these nanoparticles. They were further characterized by an absence of tumor cells, lesion and oedema, supporting the idea that the treatment leads to full tumor disappearance without inducing severe side effects. We conclude that although the tumor temperature stops increasing following the fifth MS (Fig. 5a) and magnetosome sizes are significantly reduced during the course of the various treatments (Figs. 3 and 4), anti-tumor activity remains persistent.

### Possible mechanisms explaining anti-tumor efficacy under conditions of weakened nanoparticle heating power and nanoparticle size reduction

**Fig. 7:**
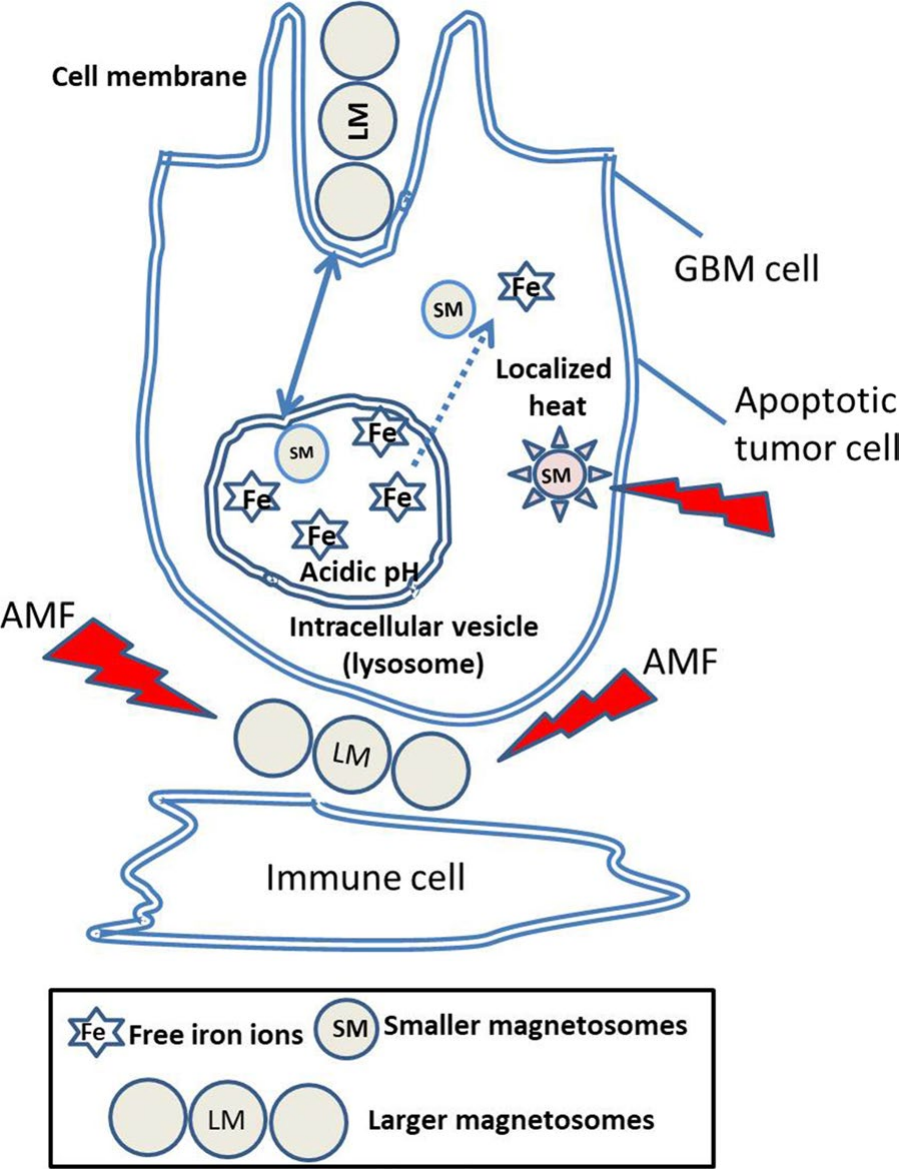
Suggested mechanisms of tumor cell destruction by magnetosomes exposed to an alternating magnetic field, involving: (i) magnetosome cellular internalization, (ii) localized heat production, (iii) apoptosis of tumor cells, and (iv) attraction of immune cells that could act against the tumor

Figure 7 summarizes the different anti-tumor mechanisms that could occur under conditions of no/moderate heating:

- An immune reaction against the tumor that could be due to endotoxins being released from the magnetosomes following AMF applications, as partly highlighted elsewhere by showing the presence of poly-nuclear neutrophils (PNN) in the magnetosome region following one or three MS [16], without however showing the direct involvement of PNN in the anti-tumor activity, this topic remaining a matter of controversy and open discussions [26, 27],
- Magnetosome internalization, which could favor intracellular heating or release of free iron, e.g. following capture of the magnetosomes by lysosomes as reported elsewhere, [28], hence resulting in an enhanced toxicity towards tumor cells.
- A tumor cell death mechanism dominated by apoptosis, given that apoptosis is known to occur more frequently than necrosis when cells are prone to moderate stresses and/or mild temperature increases [29].

## Experimental section

### Preparation of magnetosomes

To synthetize CM, we purchased *Magnetospirillum magneticum* strain AMB-1 magnetotactic bacteria from the ATCC (700,264). 4 × 10^7^ of these bacteria were introduced into one litter of sterile 1653 ATCC culture medium. The media containing the bacteria were then placed in an incubator at 30 °C for 7 days to enable bacterial growth and magnetosome production. After 7 days, the media were centrifuged at 4000*g* for 45 min. The bacterial pellet was washed using 1 ml of sterile water. Magnetotactic bacteria were concentrated using a strong Neodymium magnet (0.6 Tesla), re-suspended in 0.05 M TRIS and sonicated continuously with finger at 0 °C during 2 h at 30 W. The suspension of magnetosomes was washed several times with sterile water using a magnet to isolate magnetosome chains from the supernatant containing cellular debris and residual bacteria until cellular debris have disappeared from the supernate. Between each wash, sonication was carried out at 30 W by a series of three pulses of 2 s. Magnetosome chains were then re-suspended in 1 ml of sterile water. For intracranial injections, magnetosome chains were re-suspended in a sterile injectable solution containing 5% of glucose and exposed to irradiation of a UV lamp (UV) for 12 h for partial sterilization.

### TEM

To determine nanoparticle sizes, shapes, and organization, 7 µl of nanoparticle suspension were deposited on top of a carbon grid, left to dry, and then nanoparticles were then imaged using a transmission electron microscope (JEM-2100, JEOL, Japan). To obtain TEM micrographs of assemblies of cells and nanoparticles, we prepared the samples in the following manner: (i), removal of culture medium from the sample containing U87 cells incubated with magnetosomes for 24 h, (ii) washing of cells with 0.2 M sodium cacodylate buffer, (iii) fixing cells for 1 h at room temperature with 2.5% glutaraldehyde in 0.2 M sodium cacodylate buffer, (iv) washing two times the cells with 0.2 M cacodylate buffer, (v) storing the cells at 4 °C, (vi) post-fixing of cells with osmium tetraoxide 1% and passing cells through uranyl acetate, (vii) dehydrating cells in an ethanol series (30–100%), (viii) embedding cells in epoxy medium (EPON 812; Shell Chemical, San Francisco, California), (ix) cutting in thin sections with a microtome. Ultrathin Sections (80 nm) were stained by lead citrate and were examined by using a ZEISS EM902 TEM operated at 80 kV (Carl Zeiss-France, MIMA2 Microscopy Platform, UR1196, INRA, Jouy en Josas, France). Images were acquired with a charge-coupled device camera (Megaview III) and analyzed with ITEM Software (Eloïse, France).

## Materials and method

### ICP-AES

Iron concentrations of the samples was then determined using ICP-AES measurements (ICP-AES ICAP6200 ThermoScienbtific). A natural abundance iron standard solution was analyzed during sample runs in order to account for charges in the systematic bias. The calibration curve was obtained using four iron standard solutions (Sigma-Aldrich) in the range 0.1 –0.05 ug/ml.

### Nanoparticle characterization by absorption, CHNS, FTIR, DLS, magnetic measurements

The stability of nanoparticles in suspension was estimated by measuring the variation of the optical density of nanoparticle suspensions at 1 mg/ml in iron, measured at 480 nm, within 15 min following the homogenization of the suspension. Zeta potential of the different nanoparticles in suspension was measured by Dynamic light scattering, DLS (ZEN 3600, Malvern Instruments, UK) using a suspension of 100 µg/ml in iron whose pH was adjusted between a pH 2 and 12 by using HCl and NaOH solutions. Nanoparticle FTIR spectra were recorded with a FTIR spectrometer (Vertex 70, Bruker, USA) on lyophilized nanoparticles suspensions mixed with KBr. The percentage in mass of organic material at nanoparticle surface was estimated using an elemental CHNS analyzer (Flash EA 1112, Thermo Fisher Scientific, USA). Magnetic properties of the nanoparticles were determined by measuring nanoparticle magnetization curves at room temperature between − 1 and + 1T, using a vibrating sample magnetometer (VSM3900, Princeton Measurements Corporation, USA).

### Measurement of the quantity of endotoxins contained in nanoparticle suspensions without magnetic excitation

The endotoxin concentration of 25 µl suspensions containing 0.4 mg/ml in iron of nanoparticles, designated as Q_BMS_, was measured with the LAL assay. The latter was carried out under sterile conditions using the 88282 ThermoScientific kit called *''Pierce LAL Chromogenic Endotoxin Quantitation Kit''*. We used a low nanoparticle concentration (0.4 mg/ml) to avoid interference between the nanoparticles and the LAL assay.

### Measurement of the percentage of endotoxins released by nanoparticles in suspension following magnetic excitation

2 µl of suspensions containing 40 µg in iron of nanoparticles were introduced at the bottom of a small caliper mimicking in vivo conditions and exposed (or not) to 1 MS, during which an AMF of 198 kHz and average strength 27 mT was applied during 30 min. The supernate was then isolated from the nanoparticles by centrifugation at 14,000*g* during 10 min and its endotoxin concentration was measured using the LAL assay, as described above. The percentage of endotoxins released corresponded to the ratio Q_AMS_/Q_BMS_ between the quantity of endotoxins in the supernate following one MS, Q_AMS_, and the quantity of endotoxins measured in the nanoparticle suspension before magnetic session, Q_BMS_.

### Determination of the nanoparticle specific absorption rate (SAR)

The variations of temperatures as a function of time were measured in the various treatments, during which nanoparticles in suspension, in contact with cells or in tumor brain were exposed to the AMF. The specific absorption rate was measured using the relation: SAR = (ΔT/δt)·(C_v_/X_Fe_), where C_v_ = 4.2 J/gK is the specific heat capacity of water, X_Fe_ is the nanoparticle concentration in iron expressed in g/mL, (ΔT/δt) is the initial temperature variation with time expressed in K/s.

### Cell cultivation

Human GBM cell lines (U87-MG Luc) transduced with a Luciferase gene were cultivated in Dulbecco's Modified Eagle Medium (DMEM) containing 10% fetal bovine serum (FBS) at 37 °C in the presence of 5% CO_2_. After reaching confluence, culture medium was removed using Hank's Balanced Salt Solution (HBSS). Following trypsinization at 37 °C during 5 min, cells were detached, FBS was then added to stop the action of trypsin, and cellular concentration was measured using a Malassez counting cell.

### In vitro* treatment of cells*

5 × 10^5^ U87-Luc cells were seeded at the bottom of Petri dishes of 35 mm diameter for 24 h. Nanoparticles of concentration in iron of 700 µg/ml were added (or not) and exposed (or not) to on MS, during which an AMF of 27 mT and 198 kHz was applied for 30 min. The treated assemblies of cells and nanoparticles, hence obtained, were incubated at 37 °C for an additional day. The medium containing (or not) nanoparticles was then removed and the cells were washed twice with cold PBS. Following the in vitro treatment, the percentages of living and apoptotic cells were measured using the FITC Annexin V/Dead Cell Apoptosis Kit (ThermoFisher scientific, reference: V13242). For that, 10 µl of the washed cells were loaded into the sample slide and were inserted completely into a Countess™ II FL Automated Cell Counter (Thermo Fisher scientific, reference: 15307812), which was able to detect Annexin and Propidium Iodide fluorescence emission. Following the in vitro treatments, the number of cells in the assemblies was counted by a Countess™ II FL Automated Cell Counter. Assemblies of washed cells were centrifuged, the supernatant was removed and replaced by 286 µl of HNO_3_ (70%). The treated assemblies were kept at 4 °C during 24 h to lyse cells and dissolve nanoparticles into free iron. Finally, 10 ml of filtered water were added to all treated mixtures and iron concentration was then determined using ICP-AES measurements. We deduced the average quantity of iron coming from the magnetosomes, which was internalized in each cell, using the following formula: Iron internalization (%) = 100*(Q/Q°), where Q and Q° correspond to the quantity of iron internalized per cells after and before treatment, respectively. After in vitro treatment described above, cells were also stained with Prussian blue, and observed under optical microscope to examine the presence of iron, which appeared in blue color.

### In vivo* mouse treatments*

The in vivo protocol was approved by the local animal ethics committee of the University Pierre-et-Marie-Curie (Paris, France). 6 weeks old CD-1 female nude mice of average weight 20 g were purchased from Charles River. All mice were treated and kept in an environment complying with ethical guidelines and surgery was carried out following the guidelines of the Institutional Animal Care and Use Committee (“Ethic committee Charles Darwin N°5”). Mice were fed and watered according to these guidelines and we used cervical dislocation to euthanize them when their weight had decreased by more than 20% or when signs of pain, unusual posture or prostration were observed. Mice were divided in 6 groups of 10 mice each treated as shown in Additional file 1: Table S2. For the various treatments, the mice were anesthetized with a mixture of Ketamine (100 mg/kg) and Xylazine (8 mg/kg) in isotonic solution (0.9% of NaCl). To administer the tumor cells at D-8 and the various administrations at D0 (glucose, IONP, CM), a surgical procedure was carried out. For that, the mouse heads were fixed in a stereotactic frame, a craniotomy was realized at coordinates (0.2.0) mm and the cell suspension or various treatments (glucose, IONP, CM) were administered at (0.2.2.) mm. To follow tumor size evolution, Bioluminescence intensity (BLI) emitted by living tumor cells was measured during the day preceding or following MS (one a weak). We estimated that BLI maximum signal was reached 10 min following luciferin administration and we therefore measured BLI at that time in each mouse. A relation between tumor volume and tumor BLI was established by measuring histologically tumor volumes in a series of mice euthanized at different days following tumor cell implantation and tumor BLI in living mice at the same days as those of the euthanasia [29]. The spatial temperature distribution in the tumor was recorded during each MS with an infrared camera (EasIRTM-2, Optophase) positioned 20 cm above the coil generating the AMF. We verified that the maximum temperature measured with the infrared camera was the same as that of the temperature measured with a thermocouple microprobe (IT-18, Physitemp, Clifton, USA) positioned at tumor center and we plotted the maximum temperature as a function of time during each MS. Mouse body weights were measured every day and mice were euthanized when losses in mouse body weights exceeded 20%. Mice, which were still alive at D242, were euthanized and brain sections were collected for further histological examination by the hematoxylin and eosin (H&E) staining to determine if the tumor had fully disappeared. Mouse survival times were plotted according to the Kaplan–Meier method [26]. Statistical significance of survival time between the different groups was evaluated using the log rank test. Parameters were expressed as median and p-values, relative to control group.

### Scanning electron microscopy (SEM) analysis of slides of mouse brains

Mouse brains were washed with 10% of sucrose, embedded in OTC (TissueTek), and kept at − 80 °C in bath of isopentane cooled by liquid nitrogen. Frozen sections of their brains were obtained by cryocut (10 µm), deposited on a stub, covered by a carbon layer and analyzed by scanning electron microscope (SEM-FEG Zeiss Ultra55). We obtained surface images of cells with nanoparticles from which we could measure magnetosome sizes.

## Conclusion

Cancer nano-therapies have raised a surge of interest in the medical field due to their potential larger benefit to risk ratio compared with more conventional treatments. To achieve optimal treatment outcome, nanoparticle distribution needs to be precisely controlled. On the one hand, nanoparticles should be degraded to enable their elimination by the organism. On the other hand, such alteration should not prevent persistent anti-tumor activity until full tumor disappearance. Here, we have chosen to study a certain type of nanoparticles, called magnetosome which are synthesized by magnetotactic bacteria, and possess a well-balanced size-distribution, i.e. with 58% of magnetosomes larger than 30 nm and 42% of them smaller than 30 nm (Table 1). This property enables a distinction to be made between conditions affecting the size of small and large nanoparticles and furthermore to study the impact of size variation on nanoparticle heating power. In addition, magnetosomes studied here are extracted from magnetotactic bacteria and consist of a ferrimagnetic iron oxide mineral core surrounded by a layer containing endotoxins, allowing endotoxin release under conditions of nanoparticle size variation. To take into consideration the diversity of altering conditions that can be encountered during a real in vivo treatment, we have chosen to treat magnetosomes in different ways, i.e., by internalizing them in U87-Luc tumor cells (first treatment), by administering them in intracranial U87-Luc tumors without (second treatment) or with (third treatment) 15 magnetic sessions of alternating magnetic field application. Whereas the second treatment corresponds to mild conditions of degradation, i.e. it results in the dissolution of the smallest magnetosomes yielding 76% of magnetosomes larger than 30 nm and 24% of them smaller than 30 nm (Table 1), the first and third treatments lead to a more stringent degradation, which is associated with the dissolution of the largest magnetosomes and therefore with a larger percentage of the smallest than largest magnetosomes, i.e. 64–92% of magnetosomes are below 30 nm while 8–36% of them are above 30 nm (Table 1). The most interesting result highlighted in this study relies on the observation that despite magnetosome alteration observed in vitro and in vivo and the loss of magnetosome heating power that it induces, i.e. SAR and ΔT decrease partly in vitro by 95% between before and after magnetosome internalization in U87-Cells or fully in vivo to reach SAR = 0 W/g_Fe_ and ΔT = 0 °C after the sixth magnetic session, magnetosomes are able to maintain their anti-tumor efficacy. Indeed, on the one hand, the percentage of living cells decreases from 55 to 30% between before and after the application of one MS on internalized magnetosomes and one the other hand intracranial U87-Luc tumors injected with CM followed by 15 magnetic sessions fully disappear among 50% of treated mice.

The results presented here are important in the field of nanomedicine, since they show that nanoparticulate systems can maintain anti-tumor activity after their degradation in vivo. To the author’s knowledge, whereas such type of behavior is desired to reach a sustained and long-term anti-tumor activity in the tumor environment, which is known to be degrading, it has not yet been reported.

## Supplementary information

**Additional file 1.** **Figure S1.** (a), % of carbon in a suspension of magnetosomes as measured with a CHNS. (b), Hysteresis curve of a dried suspension of magnetosomes. (c), variation of zeta potential of a suspension of magnetosomes between pH 2 and pH 12. (d), Heating curve of a suspension of magnetosomes (2 mg of magnetosomes in 100 µl of water) exposed to an AMF of 27 mT and 198 kHz during 600 seconds. **Figure S2.** For mice having received glucose without MS, or with 3 or 15 MS, variations of tumor BLI as a function of time (days) following tumor cell implantation, (a), temperature variation measured during each MS, (b), survival rate during the days following tumor cell implantation, (c). **Table S1.** Properties of untreated magnetosomes. **Table S2.** Treatment conditions of the different groups of mice. **Table S3.** Median survival day and associated p-value estimated for the different groups of treated mice.

## Data Availability

Please contact author for data requests.

## Abbreviations

AMF: alternating magnetic field
Magnetic hyperthermia: the application of an alternating magnetic field to an assembly of nanoparticles, often resulting in the production of heat
MS: a magnetic session during which magnetic hyperthermia is carried out
CM: chains of magnetosomes extracted from magnetotactic bacteria
