# Supplementary material for: Biodegraded magnetosomes with reduced size and heating power maintain a persistent activity against intracranial U87-Luc mouse GBM tumors
Source: J Nanobiotechnology. 2019 Dec 23;17:126. doi: 10.1186/s12951-019-0555-2 (PMC6929367; doi:10.1186/s12951-019-0555-2)

**ADDITIONAL MATERIAL**

**MAGNETOSOMES MAINTAIN STRONG ANTI-TUMOR ACTIVITY UNDER DEGRADING CONDITIONS IN A MODEL OF INTRACRANIAL U87-LUC MOUSE GLIOBLASTOMA TUMORS.**

**ADDITIONAL FIGURES:**

**Figure S1**: (a), % of carbon in a suspension of magnetosomes as measured with a CHNS. (b), Hysteresis curve of a dried suspension of magnetosomes. (c), variation of zeta potential of a suspension of magnetosomes between pH 2 and pH 12. (d), Heating curve of a suspension of magnetosomes (2 mg of magnetosomes in 100 µl of water) exposed to an AMF of 27 mT and 198 kHz during 600 seconds.

**Figure S2:** For mice having received glucose without MS, or with 3 or 15 MS, variations of tumor BLI as a function of time (days) following tumor cell implantation, (a), temperature variation measured during each MS, (b), survival rate during the days following tumor cell implantation, (c).

**ADDITIONAL TABLES:**

**Table S1:** Properties of untreated magnetosomes.

**Table S2:** Treatment conditions of the different groups of mice.

**Table S3:** Median survival day and associated p-value estimated for the different groups of treated mice.


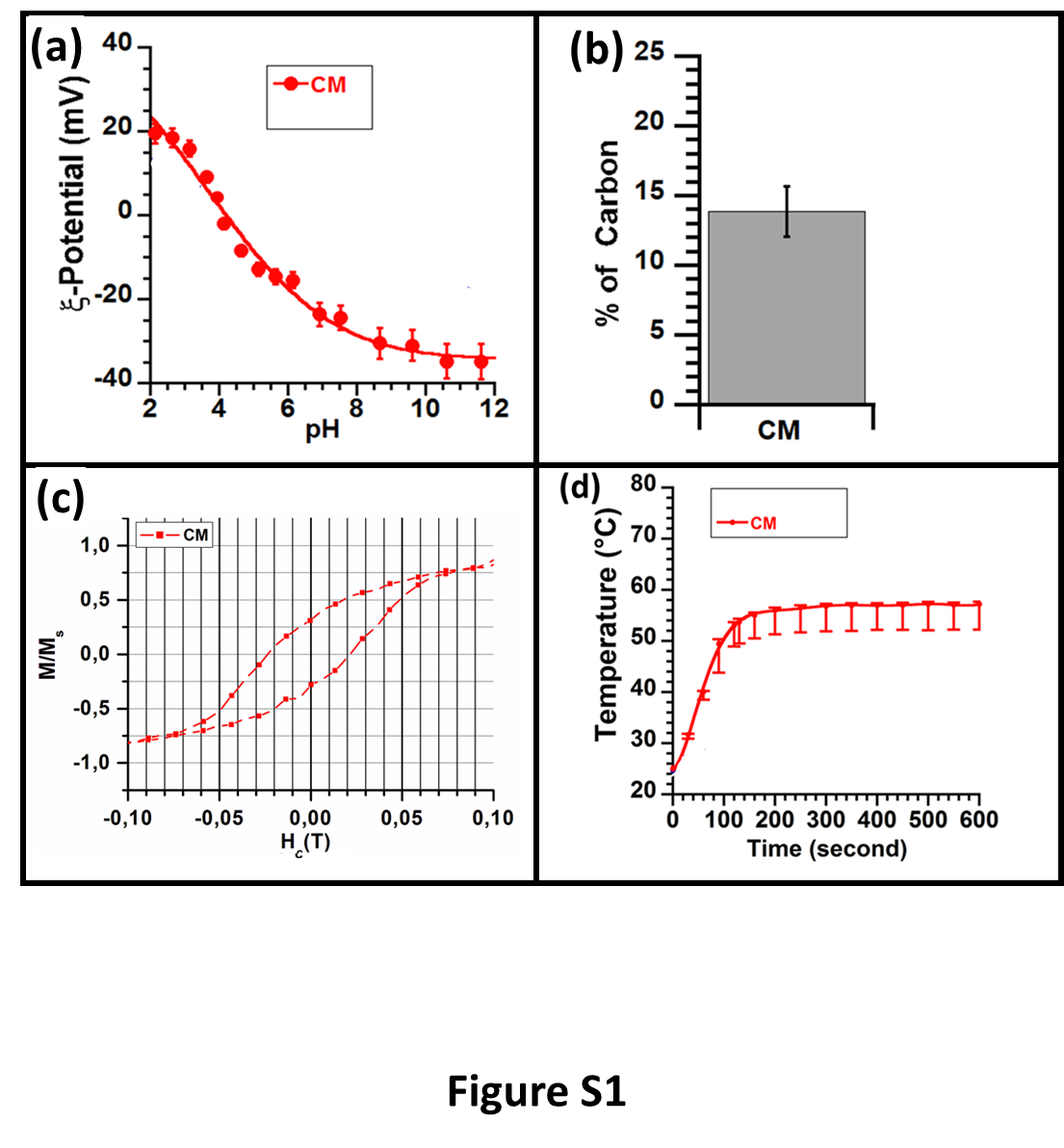


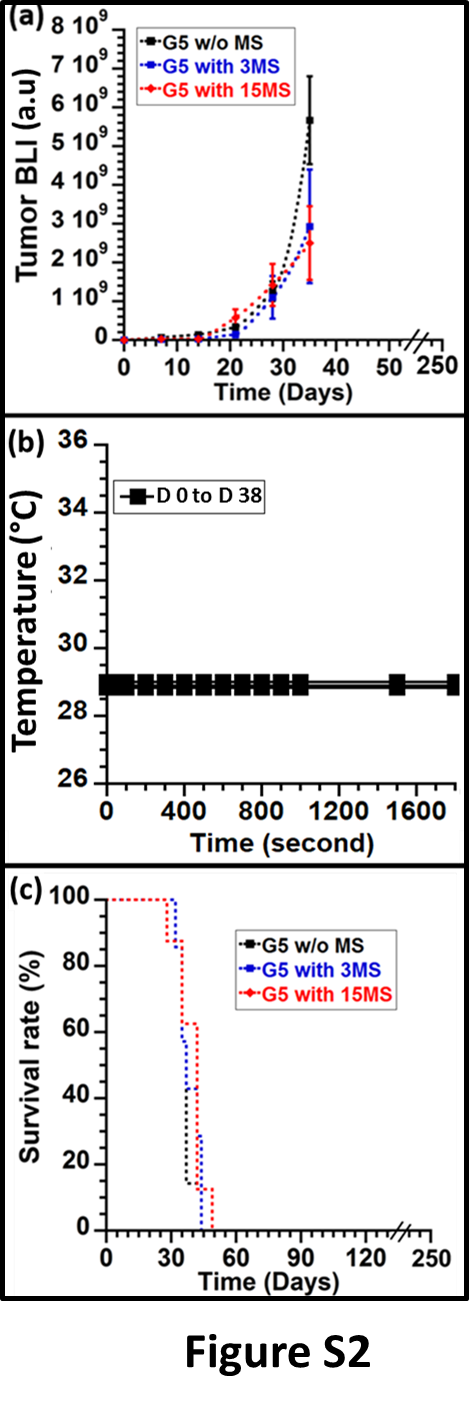


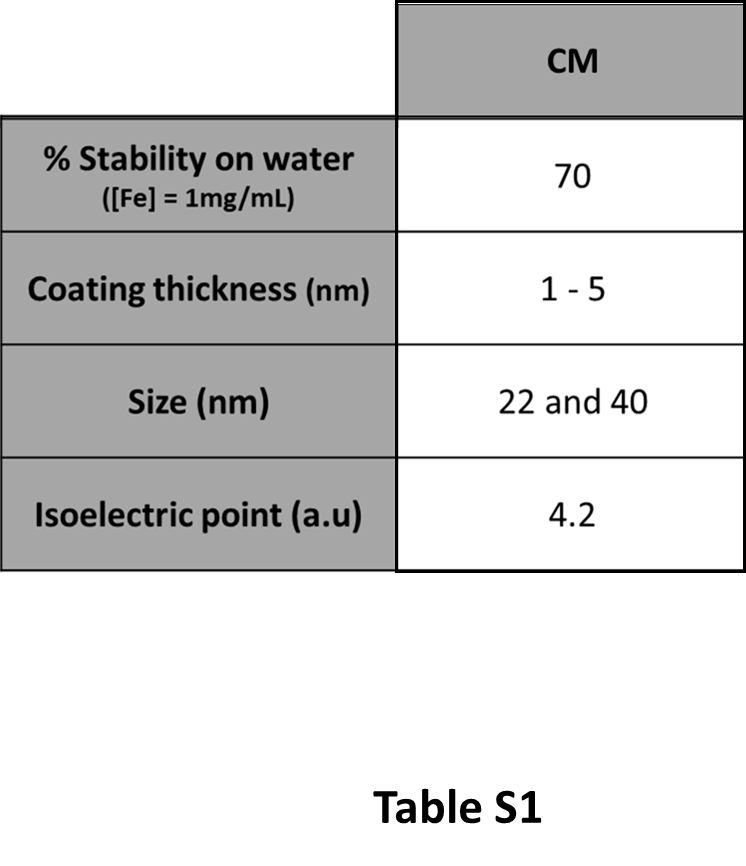


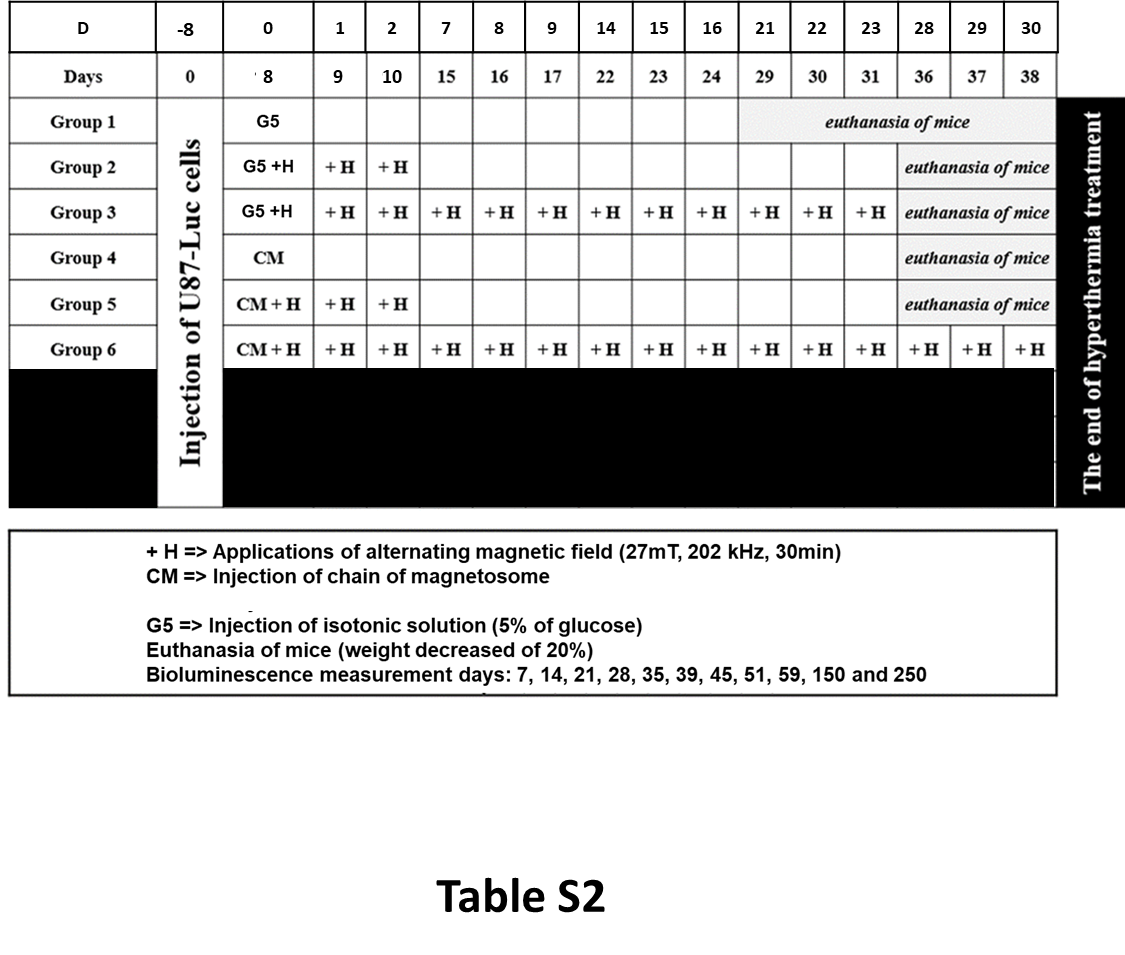


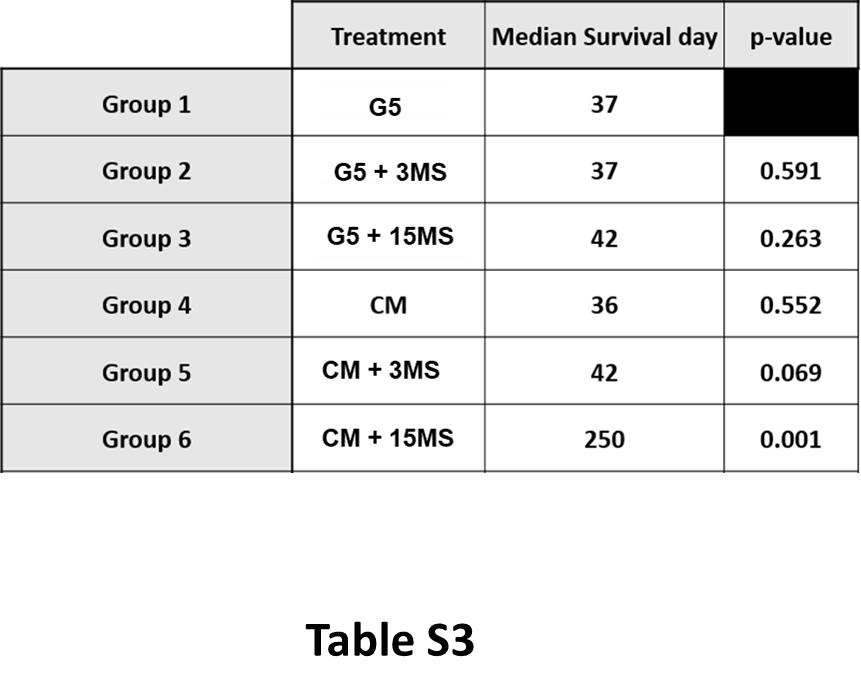

Supplement: Supplementary file 1 — Additional file 1. Figure S1. (a), % of carbon in a suspension of magnetosomes as measured with a CHNS. (b), Hysteresis curve of a dried suspension of magnetosomes. (c), variation of zeta potential of a suspension of magnetosomes between pH 2 and pH 12. (d), Heating curve of a suspension of magnetosomes (2 mg of magnetosomes in 100 µl of water) exposed to an AMF of 27 mT and 198 kHz during 600 seconds. Figure S2. For mice having received glucose without MS, or with 3 or 15 MS, variations of tumor BLI as a function of time (days) following tumor cell implantation, (a), temperature variation measured during each MS, (b), survival rate during the days following tumor cell implantation, (c). Table S1. Properties of untreated magnetosomes. Table S2. Treatment conditions of the different groups of mice. Table S3. Median survival day and associated p-value estimated for the different groups of treated mice. [file 12951_2019_555_MOESM1_ESM.docx]
